# *Dieta de la Milpa:* A Culturally-Concordant Plant-Based Dietary Pattern for Hispanic/Latine People with Chronic Kidney Disease

**DOI:** 10.3390/nu16050574

**Published:** 2024-02-20

**Authors:** Annabel Biruete, Gabriela Leal-Escobar, Ángeles Espinosa-Cuevas, Luis Mojica, Brandon M. Kistler

**Affiliations:** 1 Department of Nutrition Science, Purdue University, West Lafayette, IN 47907, USA; bmkistle@purdue.edu; 2 Division of Nephrology, Indiana University School of Medicine, Indianapolis, IN 46202, USA; 3 Departamento de Nefrología, Instituto Nacional de Cardiología Ignacio Chávez, Mexico City 14080, Mexico; leal.gabriela@hotmail.com; 4 Departamento de Nefrología y Metabolismo Mineral, Instituto Nacional de Ciencias Médicas y Nutrición Salvador Zubirán, Mexico City 14080, Mexico; angeles.espinosac@incmnsz.mx; 5 Tecnología Alimentaria, Centro de Investigación y Asistencia en Tecnología y Diseño del Estado de Jalisco (CIATEJ), Zapopan 45019, Mexico; lmojica@ciatej.mx

**Keywords:** *Dieta de la Milpa*, chronic kidney disease, dietary pattern

## Abstract

Chronic kidney disease (CKD) disproportionately affects minorities in the United States, including the Hispanic/Latine population, and is a public health concern in Latin American countries. An emphasis on healthy dietary patterns, including the Mediterranean and the Dietary Approaches to Stop Hypertension (DASH) diets, has been suggested as they are associated with a lower incidence of CKD, slower CKD progression, and lower mortality in kidney failure. However, their applicability may be limited in people from Latin America. The *Dieta de la Milpa* (Diet of the Cornfield) was recently described as the dietary pattern of choice for people from Mesoamerica (Central Mexico and Central America). This dietary pattern highlights the intake of four plant-based staple foods from this geographical region, corn/maize, common beans, pumpkins/squashes, and chilies, complemented with seasonal and local intake of plant-based foods and a lower intake of animal-based foods, collectively classified into ten food groups. Limited preclinical and clinical studies suggest several health benefits, including cardiometabolic health, but there is currently no data concerning CKD. In this narrative review, we describe and highlight the potential benefits of the *Dieta de la Milpa* in CKD, including acid-base balance, protein source, potassium and phosphorus management, impact on the gut microbiota, inflammation, and cultural appropriateness. Despite these potential benefits, this dietary pattern has not been tested in people with CKD. Therefore, we suggest key research questions targeting measurement of adherence, feasibility, and effectiveness of the *Dieta de la Milpa* in people with CKD.

## 1. Introduction

Chronic kidney disease (CKD) affects >10% of the population worldwide, with a prevalence of 15% in the United States [1]. CKD disproportionately affects racial/ethnic groups in the United States, including the Hispanic (i.e., person from a country where the primary language is Spanish) and/or Latine (i.e., nongender-based term for people from Latin America) populations [2]. Moreover, CKD is a public health concern in Latin American countries due to the high prevalence of obesity, diabetes mellitus, and hypertension [3,4].

Medical nutrition therapy provided by a registered dietitian or international equivalent is a fundamental component in the management of CKD [5]. Current nutritional recommendations for people with CKD focus on the modulation of dietary protein, phosphorus, sodium, and potassium depending on the grade of kidney dysfunction and current clinical condition [6]. In clinical practice, this nutrient-focused strategy can create dilemmas between individual nutrients (for example, phosphorus and protein [7]) and conflicting guidance that may result in confusion and poor adherence to recommendations [8,9]. An alternative strategy, which considers nutrients as a part of foods and food matrices instead of in isolation [10], is to focus on overall dietary patterns [11,12].

Healthy dietary patterns have been associated with reduced incidence, slower progression, and reduced mortality in patients with CKD; therefore, dietary patterns are a topic of ongoing research [13,14]. Among these, there has been an increased emphasis on plant-dominant patterns characterized by high consumption of vegetables, fruits, whole-grains, nuts, legumes, low-fat dairy, and minimal consumption of animal-based products and ultra-processed foods [10,13,15]. The Mediterranean and the Dietary Approaches to Stop Hypertension (DASH) diets are the most widely recognized of these dietary patterns, and the Mediterranean diet has been recommended as the dietary pattern of choice for patients with CKD [12].

However, these dietary patterns may not be widely utilized in people from Latin American countries [16]. While dietary recommendations are often not dependent on race or ethnicity, usual dietary intake and patterns may be quite different [17]. Furthermore, the lack of cultural concordance is a source of anxiety for Spanish-speaking people with CKD in the United States [18,19]. Therefore, more culturally appropriate guidance may benefit people with CKD.

A dietary pattern for Mesoamerica (Central Mexico and Central America) called *Dieta de la Milpa* or Diet of the Cornfield was recently described [20]. This dietary pattern is characterized by a diet based on the consumption of corn/maize (*Zea mays* L.), common beans (*Phaseolus vulgaris* L.), a variety of pumpkins/squashes (*Curcubita pepo maxima and moschata*), and chilies (*Capsicum annum* and *frutescens*) [20,21]. The diet is complemented by a diet high in vegetables and fruits commonly grown in the Mesoamerican region, including tomatoes (*Solanum lycopersicum* L.), starchy vegetables (such as sweet potato and yuca), other whole grains and legumes, fish, water, and avoidance of ultra-processed foods, red meat, and artificial sweeteners [20]. In addition to the dietary pattern, there is a strong emphasis on a healthy lifestyle, promoting physical activity, and overall well-being [20]. While this dietary pattern follows similar characteristics as the Mediterranean and DASH diets, it may be more culturally concordant for people from the Mesoamerica region. Therefore, the objective of this narrative review is to summarize the characteristics of the *Dieta de la Milpa* and to describe the potential benefits for patients with CKD, including benefits on acid-base balance, sources of protein, potassium and phosphorus management, modulation of the gut microbiome, inflammation, and cultural appropriateness. To explore the impact of the *Dieta de la Milpa* in CKD, we conducted a literature search on PubMed and Google Scholar until December of 2023 for published articles including terms “dieta de la milpa”, “milpa”, ”Mexican diet”, “pre-Hispanic diet”, “pre-Hispanic foods”, “Mesoamerican foods”, and “Mesoamerican diet” with ”kidney disease”, “renal disease”, and “renal failure”. As we did not identify data in CKD, we propose key research questions to evaluate adherence, feasibility, and efficacy of this dietary pattern in CKD.

## 2. Components of the *Dieta de la Milpa* Dietary Pattern

**Figure 1:**
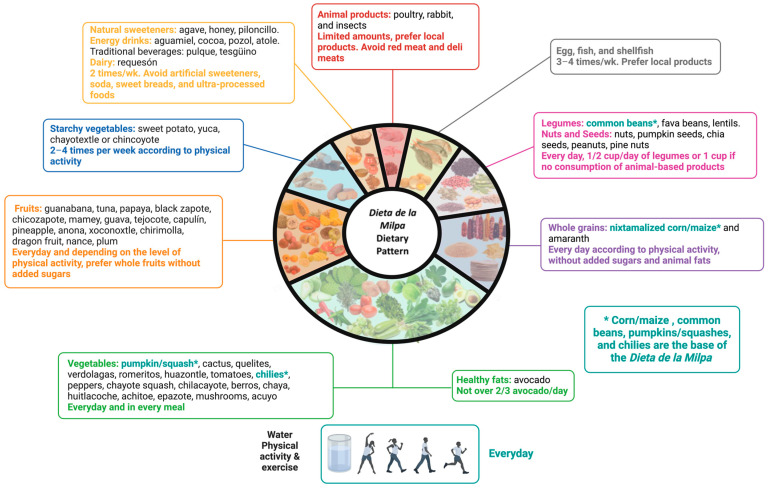
*Dieta de la Milpa* description by food groups. Adapted with permission from [20].

The origins of this dietary pattern date back to pre-Hispanic times, when *milpas* or cornfields were utilized as a productive and sustainable agricultural system to grow local seeds [20,21]. Traditionally, the main crops were corn/maize, common beans, and pumpkins/squashes, which, along with chilies, are the basis of the *Dieta de la Milpa* [20]. These four foods have important nutritional characteristics and are often the base of the traditional Mexican diet [22]. Nixtamalized corn, or corn processed with calcium hydroxide, is considered a good source of energy, dietary fiber, and calcium [20,23]. Common beans are the main source of plant-based protein in this dietary pattern and provide dietary fiber, phosphorus, potassium, and bioactive compounds [20,24,25]. There are several types of pumpkins/squashes, but zucchini squash is common, and the flower, seeds, and pulp are used in traditional cuisine and are good sources of dietary fiber, vitamins A, E, and C, magnesium, calcium, phosphorus, and iron, making it a nutrient-dense food [20]. Finally, different types of chilies provide color, flavor, and spiciness to foods [20]. As observed in Figure 1, Table 1, and Supplementary Table S1 [26,27,28], the *Dieta de la Milpa* has ten components. This dietary pattern is plant-dominant but fundamentally omnivorous, with limited consumption of poultry, fish, eggs, and dairy and an avoidance of red meat and ultra-processed foods [20].

## 3. Potential Benefits for People with CKD (Figure 2)

### 3.1. Dietary Patterns vs. Nutrient Intake

Dietary restrictions, particularly sodium, potassium, and phosphorus in moderate-to-severe grades of kidney dysfunction, have been the foundation of the nutritional approach to patients with CKD with and without kidney replacement therapy [35]. The main objective of these dietary recommendations in advanced CKD is to slow the progression of kidney disease and treat some of the complications derived from kidney dysfunction [5]. However, dietary prescription can become restrictive, and this may limit adherence [8,9]. Furthermore, dietary prescription without proper nutritional evaluation and monitoring can severely compromise the quality of the diet and the nutritional status of the patient [5,13,36].

**Figure 2:**
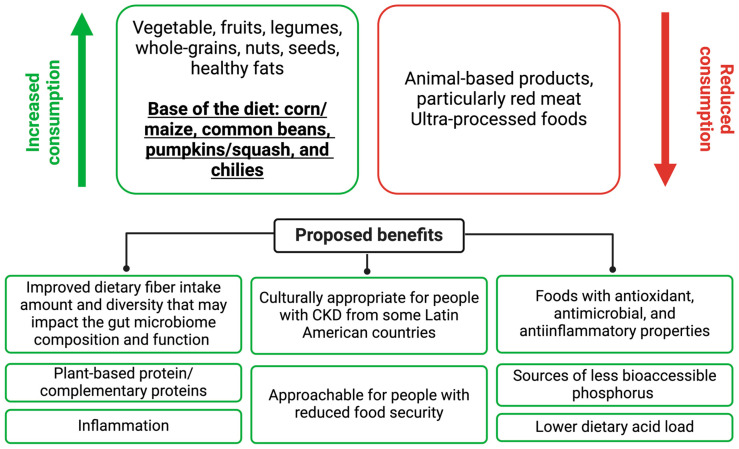
Proposed benefits of the *Dieta de la Milpa* in CKD.

An alternative to individual nutrients is to focus on dietary patterns. Healthy dietary patterns, particularly the Mediterranean and DASH diets, have been shown to confer benefits in CKD. The clinical benefit of these patterns may be explained by the impact they have on risk factors of incidence [14,37], the progression of CKD [38,39], and lower mortality [13,38]. In addition to hard outcomes, such as kidney disease progression and mortality, there may be an important benefit of healthy dietary patterns on patient-centered outcomes, including quality of life and a higher degree of satisfaction and adherence to the dietary prescription [40]. Overall, there seems to be a new perspective that the traditional renal diet needs to be revised and that a liberalized diet focused on dietary patterns may be more beneficial for patients with CKD [8,11,30].

The Mediterranean and DASH diets are plant-dominant, omnivorous dietary patterns that share many characteristics with the *Dieta de la Milpa* (Table 2). Some similarities among the three dietary patterns include a high intake of vegetables and fruits, whole-grains, legumes, and nuts, while limiting the intake of animal-based protein, sweeteners, and ultraprocessed foods. Dairy is strongly emphasized in the DASH diet, while the Mediterranean and *Dieta de la Milpa* diets recommend low-to-moderate consumption. Considering these characteristics, the *Dieta de la Milpa* may allow for greater plant-based diversity, adaptability, and eating viability by the Latin American population, including those with CKD (Table 2). However, to date, there is no clinical evidence to suggest whether the feasibility and efficacy are similar to other healthy dietary patterns.

### 3.2. Dietary Acid Load

In CKD, there is a reduced ability to excrete acids such as ammonium and titratable acids [41]. These alterations in acid-base balance increase the risk of developing acidosis and may lead to muscle wasting secondary to enhanced catabolic pathways, lower anabolism, insulin resistance, a higher risk of kidney stones, and a faster progression of CKD [41,42,43].

Diet is a significant contributor to acid-base balance in kidney disease. Nutrients can be classified as acid-forming or base precursors [41,44]. The acid-producing nutrients include phosphorus and sulfur-containing amino acids, such as cysteine, methionine, and taurine, while the base-forming nutrients include potassium, magnesium, and calcium [42,44,45]. Overall, it is considered that animal-based foods, such as meat products and ultra-processed foods are acid precursors, while fruits and vegetables are base-producers, with dairy and legumes often considered neutral [41,42]. Fruits and vegetables have been shown to correct metabolic acidosis and preserve kidney function to the same extent as bicarbonate supplementation [46,47,48]. In the *Dieta de la Milpa,* base-producing foods and neutral foods are emphasized, including vegetables, fruits, whole grains, legumes, and dairy. In contrast, animal-based foods and ultra-processed foods are recommended at lower quantities or to be avoided. These characteristics may positively impact patients with or at risk of metabolic acidosis, particularly those with CKD G3 and higher. However, trials that test the effectiveness of the *Dieta de la Milpa* in helping with acid-base balance are needed.

### 3.3. Dietary Protein Sources

Dietary protein quantity is a primary focus for patients with CKD. People with non-dialysis-dependent CKD (moderate and advanced) are recommended to consume lower dietary protein (0.55–0.6 g/kg/d or 0.28–0.43 g/kg/d with the use of keto acid analogs) to limit the progression of CKD, while people undergoing dialysis are recommended higher amounts (1.0–1.2 g/kg/d for hemodialysis and peritoneal dialysis) to account for higher requirements and losses in the dialysis treatment [6].

In addition to the protein quantity, there is a growing focus on dietary protein sources [49,50]. In the results of the ARIC cohort, Haring et al. [51] reported a high risk of incidence for CKD when there is a higher consumption of animal protein, specifically red meat and processed meat products (HR 1.23 [95% CI 1.06–1.42]; *p* < 0.01), whereas higher consumption of plant-based protein and dairy had a kidney-protective role. Additionally, in the same analyses, the daily substitution of a portion of red meat by a portion of low-fat dairy, nuts, or legumes was associated with a lower incidence of CKD, concluding that there was a strong association between the development of a CKD and the source of protein instead of just the total daily amount of protein [51].

In agreement with data demonstrating the value of considering protein source, there is growing support for plant-dominant diets [49,52,53]. However, one potential concern with the emphasis on plant-based protein, although primarily theoretical, is the potential for these patterns to worsen PEW due to the lower biological value of these proteins [54]. The *Dieta de la Milpa* emphasizes the use of complementary proteins through the combination of plant protein sources (i.e., legumes and grains), including the corn/maize + beans combination, improving the amino acid profile. Furthermore, this dietary pattern, while plant-dominant, remains omnivorous, with the consumption of dairy, eggs, fish, poultry, and insects, with a recommendation to avoid red meat. Therefore, it is plausible that the *Dieta de la Milpa* can be a dietary pattern that can be applied across the spectrum of CKD, focusing on the quantity, type, and combination of dietary protein sources, but this remains to be explored in clinical trials.

### 3.4. Phosphorus Management

CKD-MBD is a systemic disorder that is highly prevalent in patients with CKD, and its prevalence increases as kidney function declines [55]. Dietary phosphorus reduction is often recommended for patients with CKD, especially those with hyperphosphatemia [6,56]. The 2017 KDIGO guidelines recommend reducing the phosphorus intake with the objective of normalizing phosphorus [56]. Additionally, the guidelines suggest focusing on sources of dietary phosphorus and phosphorus bioaccessibility (i.e., the phosphorus available for absorption), limiting the intake of ultra-processed foods with phosphate-containing additives and preferring sources from plant-based foods, as most of the phosphorus is phytate-bound and less bioaccessible [33,56,57]. The implementation of this restriction may be challenging, as phosphorus additives are ubiquitous in manufactured foods [57].

In the *Dieta de la Milpa*, there is an emphasis on plant-based foods, which can contain most of the phosphorus in the form of phytate. Due to the limited expression of phytase, the absorption may be limited, particularly compared to phosphorus in animal-based sources and phosphate additives contained in ultra-processed foods [33]. However, it is important to note that while phosphorus may be bound to phytate, processes such as fermentation, soaking, and germination may release some of the phytate-bound phosphorus, allowing for its absorption [58]. Nonetheless, plant-based diets have been shown to reduce phosphorus excretion and circulating levels of phosphorus and fibroblast growth factor-23 (FGF23), suggesting a lower phosphorus absorption. For example, Moe et al. [59] showed that diets that contained the same amount of phosphorus but differed in the source of phosphorus (plant vs. meat) led to a lower phosphorus excretion and circulating fibroblast growth factor-23 (FGF23). Similarly, Moorthi et al. [60] showed that in people with CKD stage 3–4, a diet with 70% plant-based protein also led to lower urinary phosphorus excretion. Therefore, it is possible that the *Dieta de la Milpa* dietary pattern may reduce the risk of hyperphosphatemia and potentially reduce the need for phosphate binders [61]. However, the benefits of the *Dieta de la Milpa* on markers of CKD-MBD have not been assessed.

### 3.5. Potassium Management

Due to the central role of the kidney in maintaining potassium homeostasis, traditional dietary guidance has restricted potassium intake in people with CKD [35]. However, observational data have questioned the strength of the relationship between dietary and serum potassium, suggesting that other factors are involved in the risk for hyperkalemia [29,62,63]. As such, it is suggested to prioritize addressing possible non-dietary causes of hyperkalemia, including hyperglycemia, acidosis, constipation, recent medication changes, and use of potassium-sparing diuretics [64]. Taking this into account, recent guidelines are less restrictive in their guidance on potassium [6]. Dietary patterns like *Dieta de la Milpa* may further reduce the dietary risk of hyperkalemia by reducing the intake of potassium additives present in processed foods [65], increasing the consumption of foods with complex carbohydrates that can drive the release of insulin, increasing fiber intake which may support fecal potassium excretion [66], and by limiting transit time, especially when compared to increased intake of high-potassium and low-fiber foods such as meat [53,63]. Although concerns remain, especially related to postprandial kalemia [63], recent data supports the notion that liberalized plant-based diets can be tolerated by patients with CKD, but further research is needed [46,47,48].

### 3.6. The Gut Microbiome

Diet has been shown to modulate the composition and function of the gut microbiota [67]. Two of the major nutrients that may modulate the composition and function of the gut microbiome in CKD are dietary fiber and protein [68]. In patients with CKD, dietary fiber consumption has been reported to be below the recommendations for healthy adults [69,70], while dietary protein is often higher in patients with non-dialysis CKD [71]. This lower dietary fiber intake may lead to reduced production of short-chain fatty acids (SCFAs), products of bacterial fermentation of dietary fiber that have been often associated with positive health outcomes [72], while microbiota-derived uremic toxins, indoxyl sulfate and p-cresyl sulfate, increase with kidney dysfunction, primarily due to a reduced ability to excrete these compounds as they are mostly protein-bound [73].

Foods emphasized in the *Dieta de la Milpa* that enhance the quantity and variety of types of dietary fiber, plant-based protein, and polyphenols may have the potential to impact gut microbiota composition and function, driving host effects [70,74]. Traditional Mexican foods have been associated with improvements in the gut microbiota in experimental studies in murine models of metabolic disease [75] and recently in patients with metabolic syndrome [76]. Specifically, maize (*Zea mays* L.), nopal (*Opuntia ficus*), and beans (*Phaseolus vulgaris* L.) have food matrices that may contain beneficial bacteria, prebiotic fibers, and polyphenols that may be beneficial for the gut microbiome [77]. For example, nixtamalized corn contains prebiotic compounds such as ferulated arabinoxylans, which can promote the growth of *Bifidobacterium* and increase the production of SCFAs [77]. Corn cobs are sources of xylooligosaccharides with prebiotic properties promoting the production of SCFAs and the growth of *L. plantarum* S2, which may have antimicrobial effects limiting pathogenic bacteria and maintaining gut homeostasis [77]. Nopal (*Opuntia ficus*) contains viscous, fermentable fibers (pectins and gums) and insoluble fibers (cellulose and lignin) that may have prebiotic and bulking effects, and it was shown to shift the gut microbiota composition and enhance the production of SCFAs in rats fed a high-fat diet [78,79]. Finally, common beans (*Phaseolus vulgaris* L.) increase *Lactobacillus* and *Bifidobacterium* and contain prebiotic oligosaccharides that may increase the bioavailability of minerals, including zinc and iron [77]. While the focus has mostly been on dietary fiber and protein, plant-based diets, including the *Dieta de la Milpa*, contain a variety of known and unknown compounds that also may be metabolized by the gastrointestinal microbiota, and further investigation is warranted [80].

### 3.7. Inflammation

CKD is described by a state of chronic low-grade inflammation, with elevated levels of both pro- and anti-inflammatory cytokines, due to a combination of reduced renal clearance as well as increased cytokine production potentially due to numerous diet-related factors, including advanced glycation, carbonyl stress, fluid overload, and acidosis [81,82,83,84,85]. Healthy behaviors, including healthy dietary patterns and physical activity, have been shown to reduce chronic inflammation [12,86]. Individual nutrients and foods may also modulate chronic inflammation, including dietary fiber, *n*–3 polyunsaturated fatty acids, and foods including fruits, vegetables, and legumes [10]. Dietary patterns that incorporate the intake of these foods and reduced intake of sugar and animal fats are associated with lower markers of inflammation [14]. In the *Dieta de la Milpa*, it is common for food preparations to be accompanied by sauces that contain a combination of tomatoes, chili peppers, and onion. The main ingredients used to prepare the sauces are tomato (*Lycopersicum esculentum*), tomatillo (*Physalis ixocarpa*), cilantro (*Coriandrum sativum*), onion (*Allium cepa*), garlic (*Allium sativum*), and chili (*Capsicum annuum*). These ingredients are sources of phenolic compounds, including phenolic acids, flavonoids, and capsaicinoids, which have antioxidant, antimicrobial, anti-inflammatory, and vasodilatory properties, among others [87]. Given these observations, it may be hypothesized that the *Dieta de la Milpa* could positively affect inflammation in people with CKD.

### 3.8. Cultural Appropriateness

One of the caveats with general nutritional recommendations for kidney disease is that these do not consider the diverse dietary patterns of people with CKD across the world. For example, countries that have a higher prevalence of plant-based eating patterns may have been at odds with previous nutritional recommendations due to the high intake of dietary potassium and total dietary phosphorus, without taking into consideration their effects on acid-base balance, blood pressure regulation, and differences in bioavailability. Similarly, the traditional Mexican and Central American diet, high in plant-based foods complemented with some animal products, may have been discouraged in individuals with CKD, again likely due to the high intake of total potassium and phosphorus [18]. However, the more recent positive narrative around plant-based dietary patterns has shifted, with the focus now on overall healthy dietary patterns, and this may provide an opening venue for the incorporation of culturally appropriate dietary patterns, such as the one described in the current manuscript [19].

Food intake relates to cultural identity and goes beyond the need to fulfill physiological needs. As nutrition professionals, we understand that what an individual eats goes beyond the nutrition prescriptions intended for CKD and that factors such as culture and the social environment impact an individual’s eating pattern and relates to satisfaction and adherence. Hence, integrating and promoting culturally appropriate dietary patterns within our practice may lead to better adherence and outcomes in patients with CKD [19]. This has been shown in other clinical populations, such as diabetes, but remains to be explored in CKD [88,89].

## 4. Key Research Questions to Evaluate the Measurement, Feasibility, and Effectiveness of the *Diet a de la Milpa*

While there may be theoretical benefits of the use of the *Dieta de la Milpa* due to the similarities with other healthy dietary patterns, research should focus on enhancing our understanding and evidence for the applicability of this dietary pattern in the context of CKD. We propose to focus on three main areas: (1) measurement of the dietary pattern, (2) evaluation of the feasibility and acceptability, and (3) evaluation of the effectiveness at improving clinical and patient-centered outcomes.

### 4.1. The Development or Adaptation of a Metric to Evaluate the Adherence to the Dieta de la Milpa

Cross-sectional and epidemiological studies provide important evidence to support the use of a specific dietary intervention. For the Mediterranean [90] and DASH [91] diets, scoring systems can be utilized and adapted for measuring dietary intake. Santiago-Torres et al. [92] developed a traditional Mexican diet score, which includes 12 food components (corn tortillas, beans, soups, Mexican mixed dishes, vegetables, whole fruits, rice, full-fat milk, full-fat Mexican cheeses, and low consumption of oils, solid fats, added sugars, processed meats, and refined grains), similar to the recommendations of the *Dieta de la Milpa*. Research should focus on the utilization of this traditional Mexican diet score or the development of a *Dieta de la Milpa*-specific diet score and outcomes related to CKD.

### 4.2. Evaluation of the Feasibility and Acceptability of the Dieta de la Milpa in People with CKD

Semi-structured interviews have highlighted the negative consequences of the traditional renal diet in people from Latin America [18]. However, there is no evidence of the acceptability of this dietary pattern by people affected with CKD (in the Mesoamerica region and elsewhere) as well as the feasibility of *Dieta de la Milpa* pattern consumption in countries outside of the Mesoamerica region. Moreover, while the four foundational foods (corn/maize, common beans, pumpkins/squash, and chilies) may be accessible outside the Mesoamerica region, several of the plant-based foods recommended may not. Therefore, the adaptability of the dietary pattern should also be explored.

### 4.3. Evaluation of the Effectiveness of the Dieta de la Milpa in CKD

There is evidence that a traditional Mexican diet can have cardiometabolic benefits [76,93]. However, the impact of consuming this dietary pattern on clinical and patient-centered outcomes is needed. Outcomes of interest include cardiometabolic health, kidney function, the gut microbiome, and quality of life.

## 5. Conclusions

The traditional renal diet is highly restrictive. Focusing on dietary patterns rather than the restriction of single or multiple nutrients may improve adherence across the CKD spectrum. However, commonly recommended dietary patterns, such as the Mediterranean diet, may limit foods that are staples consumed by people from Latin America and may lack cultural concordance. The recently proposed *Dieta de la Milpa* has many components that may benefit people with CKD while utilizing foods commonly utilized in this population. As this dietary pattern is plant-dominant yet omnivorous, there are plausible benefits that include improvement/maintenance of kidney function, the promotion of base-forming foods helping with acid base balance, improvement of the gut microbiome due to a higher quantity and diversity of dietary fiber, consumption of less bioaccessible phosphorus food sources helping with phosphorus management, consumption of foods with anti-inflammatory properties, and the benefit of being a culturally appropriate dietary pattern for people from the Mesoamerica region. These proposed benefits, however, currently lack evidence in the CKD context and should be tested in clinical trials to evaluate the feasibility and effectiveness of the adoption of the *Dieta de la Milpa* dietary pattern.

## Figures and Tables

**Table 1:** Components of the *Dieta de la Milpa*.

Component	Examples [20]	Characteristics and Recommendations [20]	Considerations for People with CKD
(1) Vegetables	Nopal (cactus), tomatoes, green beans, quelites, quintile, verdolagas (purslane), romeritos, huazontle, green tomatoes, chilies, bell peppers, squash, chayote squash, chilacayote, colorines, izote flower, jicama, watercress, chaya, huitlacoche, achiote, epazote, vanilla, Mexican pepperleaf, mushrooms, and others.	Eat a combination of these daily in high quantities; make them the base of your plate. Nutrient-dense foods, high in dietary fiber, vitamins, and minerals.	Consider potassium content per portion and cooking methods to reduce potassium content [29]. Consider the use of potassium binders, as described by Sussman et al. [30] in order to liberalize the diet in those at risk of hyperkalemia.
(2) Starchy vegetables	Sweet potatoes, yuca, chayotextle.	2–4 times per week considering physical activity. Combine them with legumes and vegetables	Consider potassium content per portion [29]. Consider carbohydrate content and its impact on insulin release, as this may limit the rise in serum potassium [29,31,32].
(3) Fruits	Guanabana (soursop), tuna, papaya, black zapote, chicozapote, mamey, guava, tejocote, capulin, pineapple, anona, xoconostle, chirimoya (custard Apple), nance, berries, yellow plum, dragon fruit.	Consume daily according to physical activity. These foods are high in dietary fiber, vitamins, minerals, antioxidants and should not be consumed with added sugar. Whole fruit consumption is recommended rather than juice.	Consider potassium content per portion [29]. Consider carbohydrate content and its impact on insulin release, as this may limit the rise in serum potassium [29,31,32]. The objective is to maintain serum potassium < 5.5 mmol/L (ideal < 4 mmol/L) [32].
(4) Legumes and (5) nuts	Common beans, lima beans, squash seeds (pepitas), chickpeas, lentils, chia seeds, peanuts, and pine nuts.	Consume daily ½ to 1 cup, prioritize daily consumption if animal-based proteins are not consumed. These foods are high in plant-based proteins, dietary fiber, iron, fat, and B-vitamins.	Consider cooking methods to reduce potassium and phosphorus in beans [29,33]. Consider portion control [29,33].
(6) Healthy fats	Avocado.	Not more than ¾ of an avocado daily. High in dietary fiber, potassium, vitamin E, vitamin C, and monounsaturated fats.	Consider potassium content per portion [29].
(7) Whole grains	Corn/maize, amaranth.	Daily according to physical activity; consider sources without added sugars and animal-based fats. High in energy, dietary fiber, calcium, iron, folic acid, phosphorus (phytate-bound), potassium.	Consider potassium content per portion [29]. Consider carbohydrate content and its impact on insulin release, as this may limit the rise in serum potassium [29,31,32].
(8) Animal protein a. Eggs and seafood b. Poultry c. Insects	Eggs, catfish, trout, white fish, bass, mojarra, sierra, crab, mussels, oysters, acamayas, octopus, shrimp. Local chicken and turkey. Crickets, maguey worms, chinicuiles, chicatana ant, honey ant, jumiles.	With poultry and insects, 3–4 times per week. Moderate consumption of these foods is recommended. Combine with vegetables. Sources of protein and phosphorus. Insects are also sources of dietary fiber.	Consider protein depending on the stage of CKD [6]. Limited research on insects in CKD.
(9) Dairy	Requesón	≤2 portions per week. Source of protein, phosphorus, calcium, and probiotic strains.	Consider sodium, potassium, and phosphorus content per portion [8,29,33].
(10) Honey and sweeteners	Honey, agave nectar, piloncillo.	Not more than 2 teaspoons of piloncillo or honey in healthy individuals.	Consider sugar content [34].
Water and fermented beverages	Water, pozol, aguamiel of maguey, chocolate, tesgüino.	Prefer water consumption. Limit consumption of fermented beverages high in sugar.	Consider sugar content [34].

**Table 2:** Advantages and Disadvantages of Dietary Patterns in Chronic Kidney Disease.

	Characteristics	Advantages	Disadvantages
Mediterranean Diet	High consumption of fruit, vegetables, legumes, nuts, whole grains, olive oil and fish Low-to-moderate consumption of dairy Low consumption of ultra-processed foods, saturated fats, red meat, and poultry Regular consumption of wine Key nutrients: monounsaturated and polyunsaturated fatty acids, potassium, and dietary fiber Low consumption of sodium	The main sources of protein are plant-based proteins and white meats Low consumption of red meat and ultra-processed foods Lower consumption of sodium, inorganic phosphorus, and added potassium Evidence of improvement in endothelial function, lipid profile, inflammatory parameters, and blood pressure Dietary pattern of choice for CKD	It does not take Mesoamerican food items into consideration, which may increase costs for Latin American countries Not designed to reduce the progression of CKD, resulting in some authors suggesting the customization of eating patterns to avoid the risk of hyperkalemia as a result of a high intake of fruits and vegetables
DASH Diet	High consumption of fruits, vegetables, and low-fat dairy Low consumption of ultra-processed foods, saturated fat, sodium, artificial sweeteners, and other added sugars Key nutrients: potassium, calcium, magnesium, and dietary fiber Low consumption of sodium	High consumption of dietary fiber and potassium Higher consumption of plant-based products with suggested lower potential renal acid load Evidence of improvement in blood pressure, lipid profile, and cardiovascular risk	It does not take Mesoamerican food items into consideration, which may increase cost for Latin American countries Not designed to reduce CKD progression, resulting in some authors suggesting the customization of eating patterns to avoid the risk of hyperkalemia as a result of plant-based food consumption
*Dieta de la Milpa*	Base of the diet: corn/maize, common beans, pumpkins/squashes, and chilies High consumption of fruits and vegetables Moderate consumption of dairy and natural sweeteners Low consumption of ultra-processed foods and red meat Key nutrients: potassium, dietary fiber, and organic sources of phosphorus Low consumption of sodium	Fits regional and economic environment by incorporating local food items Viable eating pattern, even for patients with food/nutrient insecurity No studies assessing its efficacy on potential cardiovascular, metabolic, and kidney-related outcomes	Observational or intervention studies to evaluate the impact on patients with CKD are not available

## Supplementary Materials

The following supporting information can be downloaded at: https://www.mdpi.com/article/10.3390/nu16050574/s1, Table S1: Nutrient composition of some foods recommended in the *Dieta de la Milpa* food pattern.
