# Supplementary material for: Dieta de la Milpa: A Culturally-Concordant Plant-Based Dietary Pattern for Hispanic/Latine People with Chronic Kidney Disease
Source: Nutrients. 2024 Feb 20;16(5):574. doi: 10.3390/nu16050574 (PMC10934134; doi:10.3390/nu16050574)
Supplement: Supplementary file 1 [file nutrients-16-00574-s001.zip › nutrients-2857491-supplementary.pdf]

Supplementary Table S1. Nutrient composition of some foods recommended in the *Dieta de la Milpa* food pattern.

Food	Energy	CHO	Fat	Protein	Dietary fiber	Sodium	Potassium	Phosphorus	Suggested serving size &
	Kcal/100g	g/100g				mg/100g			
Pumpkin*	20	3.55	0.26	1.62	0.43	-	-	-	½ cup. cooked (107g)
Squash (zucchini)*	16	2.88	0.30	2.03	1.00	8	261	44.50	1 piece (~90g)
Squash (creole)*	18	3.17	0.05	1.80	0.30	-	-	24.00	1 piece (79g)
Squash (yellow)*	20	3.48	0.06	1.86	2.10	-	-	8.00	1 piece (~90g)
Zucchini flower&	15	3.28	0.15	1.04	0.89	5..97	106	34.32	1 cup. cooked (134g)
Cactus (nopal)*	24	5.10	0.26	1.45	2.20	21	257	23.50	1 cup. cooked (149g)
Quelites (papaloquelite)*	17	2.90	0.32	1.78	0.92	-	-	31	80g raw
Verdolagas*	21	2.38	0.47	2.20	0.99	13.70	793.78	25.21	1 cup. cooked (115g)
Romeritos&	36	4.90	0.19	3.60	1.00	-	-	18	1 cup. raw (120g)
Tomatoes*	13	1.30	0.12	0.69	1.10	5.00	237	24.67	1 piece (116g)
Tomatillo#	21	4.60	0.20	1.20	1.10	13	204	20	5 pieces (86g)
Chili (raw)#	38	8	0.60	1.90	-	7	340	-	-
Chayote squash*	19	4.51	0.13	0.82	1.70	2	125	18	½ cup (80g)
Chilacayote*	23	5.02	0.32	1.13	0.33	-	-	30	1.5 pieces (135g)
Berros*	24.50	2.89	0.56	2.90	2.90	41	330	62.67	2 cups (56g)
Chaya*	58	6.71	1.92	7.22	2.25	-	-	58.50	3 cups (147g)
Huitlacoche*	29	6.24	0.43	1.62	1.81	-	-	39	1/3 cup cooked (66g)
Achiote*	266	55.54	3.96	14.20	13.80	-	-	220	¼ tbsp (3.75g)
Epazote*	25	4.72	0.20	2.73	5.40	43	633	42.50	½ branch (1g)
Mushrooms*	29	4.58	0.11	4.69	0.60	6	448	76	½ cup. cooked (78g)
Sweet potatoes*	103	27.56	0.75	0.85	3.00	55	337	34	1/3 cup (69g)
Yuca*	116	28.25	0.59	1.00	1.80	14	271	32	¼ piece (60g)
Chayotextle/chincoyote*	139	5.14	12.63	1.30	0.65	327.37	130.27	-	-
Guanabana*	66	16.84	0.30	1.00	4.30	14	278	27	1 small piece (238g)
Tuna (fruit)*	44	10.24	0.58	0.84	4.50	5	220	32	2 pieces (138g)
Papaya*	33	8.54	0.07	0.52	1.10	8	182	6.50	1 cup (140g)
Black zapote*	56	14.46	1.10	0.60	5.30	12	193	28.75	½ piece (93g)
Chicozapote*	73	17.40	1.39	0.50	5.30	12	193	8	½ piece (75g)
Mamey*	70	16.25	0.26	1.93	5.90	15	47	31.30	1/3 piece (85g)
Guava*	46	15.57	0.47	0.98	5.40	2	417	28.60	3 pieces (124g)

Tejocote*	86	21.99	0.55	0.78	2.74	-	-	32.50	2 pieces (60g)
Capulin*	68	16.76	0.05	1.52	0.64	-	-	25.67	3 cups (96g)
Pineapple*	33	8.37	0.12	0.56	1.20	1	109	12	¾ cup (124g)
Anona*	96	25.20	0.20	2.34	2.40	4	382	20	130g
Xoconostle*	22	5.20	0.36	0.09	4.55	-	-	16	3 pieces (72g)
Chirimoya*	64	14.31	0.37	2.38	3.00	7	287	27	1/3 piece (56g)
Nance*	49	11.70	1.35	0.87	2.55	-	-	14	642 g
Berries*	23	5.28	0.17	0.84	2.30	1	153	32	¾ taza (108g)
Yellow plum*	46	11.42	0.42	0.61	1.40	0	157	24	3 pieces (158g)
Dragon fruit*	54	13.55	0.11	1.44	2.10	-	-	15	2 pieces (108g)
Beans (cooked)*	75.50	12.89	0.37	5.90	5.49	226	272	127	½ cup. cooked (86g)
Lima beans (dry)*	341	58.29	1.53	22.60	25	13	1062	439	½ cup. cooked (85g)
Pepitas (salted)*	455.10	7.80	35.90	25.20	20.20	1727.40	804.16	266.90	60 pieces (12g)
Chickpeas (cooked)*	118	21.10	0.39	8.34	5.60	2	362	99	½ cup (120g)
Lentils (dry)*	329	56.53	1.64	22.73	30.50	6	955	318.67	½ cup (99g)
Chia seeds#	490	43.85	30.75	15.62	37.70	19	160	948	5 tbsp (11.65g)
Peanuts (raw)*	519	19.34	38.96	26.95	8.50	16.75	634.52	362.33	14 pieces (12.25g)
Pine nuts*	673	13.08	68.37	22.40	3.70	2	597	588	1 tbsp (10g)
Avocado*	42	4.82	24.50	1.69	4.70	7	485	48	1/3 piece (31g)
Corn/maize nixtamalized*	375	74.16	4.56	8.95	9.60	7.73	263	214	2.5 tbsp flour (17.81g)
Amaranth (grain)*	366	65.50	6.13	13.38	6.70	0	537.98	572.67	¼ cup. roasted (16.25g)
Eggs*	145	4.93	8.62	12.30	0	133.06	115.17	192.03	1 piece (50g)
Catfish* (raw)*	119	0	1.93	15.23	0	98	302	204	80g. raw
Trout (raw)*	94	0	1.67	18.49	0	52	361	122	70g. raw
Bass (raw)*	94	0	1.67	20.02	0	70	356	204	75g. raw
Mojarra (raw)*	106	0	2.67	19.16	0	68	256	260	35g. raw
Crab (raw)*	72	0	0.97	14.85	0	62	261	218	2 pieces (42g). raw
Octopus (raw)*	57	2.20	0.32	12.61	0	230	350	109	45g. raw
Oyster (raw)*	39	1.10	1.00	6.00	0	65.60	168	162	75g. raw
Shrimp (raw)*	102.91	4.40	1.59	17.69	0	71.87	265.37	197.15	6 pieces. medium (36g)
Atole (powder)*	399	78.12	5.26	8.58	0.20	-	-	309.50	7 tbsp (18g)
Aguamiel of maguey*	282	68.53	0.67	2.23	0.10	-	732.81	110.19	-
Chocolate*	363	14	2.20	72.80	15	19	397	287	13g
Honey*	336	86.27	0.48	0.27	0.20	4	52	4	2 tsp (14g)
Piloncillo#	377	97.33	0	0	0	39	346	22	10g
Requesón*	86	10	0.85	13.80	0	450	54	134	3.5 tbsp (42g)
Chicken*	234	1.10	18	15.80	0	21.67	173.27	147	40g. raw
Turkey*	123	0.30	3.40	22.80	0	67	269	180	45g. raw
Crickets									
Grasshoppers&	637	21.90	14.90	63.09	11.36	0.90	-	-	11g

Maguey worms&	200	0	13.68	16.68	0	-	-	-	35g
Jumiles	446	0	34.20	32.20	0	-	-	-	20g

*[27]; #[28]; &[29]

References

27. Instituto Nacional de Ciencias Médicas y Nutrición Salvador Zubirán. Tables of composition of Mexican foods and food products (condensed version; 2015); Departamento de Ciencia y Tecnología de los Alimentos: Ciudad de México, México, 2016.

28. 27. Instituto de Nutrición de Centro América y Panamá (INCAP) y Organización Panamericana de la Salud (OPS). Tabla de composicion de alimentos de Centroamérica; INCAP/OPS: Guatemala, 2007.

29. 28. Perez-Lizaur, A.B.; Palacios-Gonzalez, B. Sistema Mexicano de Alimentos Equivalentes, 5th ed.; Fomento de Nutrición y SaludÑ Mexico City, Mexico, 2022.
